# circHECTD1 facilitates glutaminolysis to promote gastric cancer progression by targeting miR-1256 and activating β-catenin/c-Myc signaling

**DOI:** 10.1038/s41419-019-1814-8

**Published:** 2019-08-02

**Authors:** Juan Cai, Zhiqiang Chen, Jinguo Wang, Junfeng Wang, Xianjun Chen, Linhu Liang, Min Huang, Zhengrong Zhang, Xueliang Zuo

**Affiliations:** 1 Department of Oncology, The First Affiliated Hospital, Yijishan Hospital of Wannan Medical College, Wuhu, 241001 China; 2 Key Laboratory of Non-Coding RNA Transformation Research of Anhui Higher Education Institution (Wannan Medical College), Wuhu, 241001 China; 3 Hepatobiliary Center, The First Affiliated Hospital of Nanjing Medical University, Key Laboratory of Liver Transplantation, Chinese Academy of Medical Sciences, NHC Key Laboratory of Liver Transplantation, Nanjing, 210029 China; 4 Department of Gastrointestinal Surgery, The First Affiliated Hospital, Yijishan Hospital of Wannan Medical College, Wuhu, 241001 China

**Keywords:** Gastric cancer, Gastrointestinal cancer

## Abstract

Circular RNAs (circRNAs) have emerged as crucial regulators of human cancers. Glutaminolysis supplies cancer cells with adequate nitrogen and carbon to replenish the tricarboxylic acid cycle, contributing to the survival and progression of tumor cells. However, the association between circRNAs and glutaminolysis remains unclear. In this study, we showed that circHECTD1 expression was markedly upregulated in gastric cancer (GC) and was associated with lymph node metastasis and American Joint Committee on Cancer stage. The circHECTD1 expression level was found to be an independent prognostic factor for GC patients. circHECTD1 knockdown inhibited GC cell glutaminolysis, proliferation, migration, and invasion, whereas circHECTD1 overexpression promoted GC progression. Dual-luciferase and RNA immunoprecipitation assays demonstrated that miR-1256 was a direct downstream target of circHECTD1. circHECTD1 targeted miR-1256 and subsequently increased the expression level of USP5. The circHECTD1/miR-1256/USP5 axis exerted its tumor-promoting effects by activating the downstream β-catenin/c-Myc signaling pathway. In vivo mouse models further verified the oncogenic roles of circHECTD1 in GC. Our results revealed that circHECTD1 is a glutaminolysis-associated circRNA that promotes GC progression. The circHECTD1/miR-1256/USP5 axis could thus be used as a therapeutic target for GC.

## Introduction

Gastric cancer (GC) is the fifth most commonly diagnosed cancer and the third leading cause of cancer mortality worldwide, with ~1,000,000 new cases and 783,000 deaths annually^1^. Despite considerable advances in GC early diagnosis and therapeutic approaches, the prognosis of this disease remains poor. It is thus necessary to understand the underlying molecular mechanisms responsible for GC progression and identify novel therapeutic targets.

Glutaminolysis is a hallmark of cancer metabolic reprogramming^2^. Cancer cells are highly dependent on glutamine for their survival and proliferation. Accelerated glutamine uptake requires the involvement of alanine, serine, and cysteine-preferring transporter 2 (ASCT2), which is a high-affinity glutamine importer in cancer^3^. Glutamine is subsequently converted to glutamate by the rate-limiting enzyme glutaminase 1 (GLS1) and then transformed into α-ketoglutarate (α-KG)^4^. Glutamine serves as a critical nitrogen and carbon donor to replenish the tricarboxylic acid cycle. Glutaminolysis is correlated with the β-catenin/c-Myc signaling pathway and facilitates tumor progression^5,6^. Inhibition of glutaminolysis has emerged as a metabolic target for cancer therapy^7^. It has been reported that glutaminolysis plays critical roles in GC progression^8,9^; however, the underlying mechanisms are still unclear.

Circular RNAs (circRNAs) represent a novel class of noncoding RNAs that are covalently linked to form a closed loop, with no 5′ caps or 3′ poly(A) tails. Generated by back-splicing, circRNAs are resistant to exonucleases and more stable than linear splicing products^10^. circRNAs can regulate gene expression through various mechanisms, including targeting microRNAs (miRNAs), interacting with RNA-binding proteins, and functioning as translation templates^11^. To date, multiple functional cancer-associated circRNAs have been identified^12,13^. In lung cancer, has_circ_0020123 competitively binds with miR-144 to exert its oncogenic effects^14^. Through targeting miR-1271, circ-ABCB10 promotes breast cancer carcinogenesis^15^. circFAT1(e2) inhibits GC cell tumorigenesis by acting as a sponge of miR-548g^16^. Although the regulatory roles of noncoding RNAs in cancer metabolic reprogramming have been gradually recognized^17^, the association of circRNAs with glutaminolysis remains unclear.

In the current study, we identified that circHECTD1 was upregulated in GC tissues and cell lines. High expression levels of circHECTD1 were significantly associated with unfavorable survival outcomes. We found that circHECTD1 promoted glutaminolysis by modulating the miR-1256/USP5 axis, thereby exacerbating GC progression. These findings revealed a novel function for circRNAs in glutaminolysis and suggested that circHECTD1 might be a therapeutic candidate for GC.

## Results

### circHECTD1 is upregulated in GC and correlates with unfavorable prognosis

**Fig. 1:**
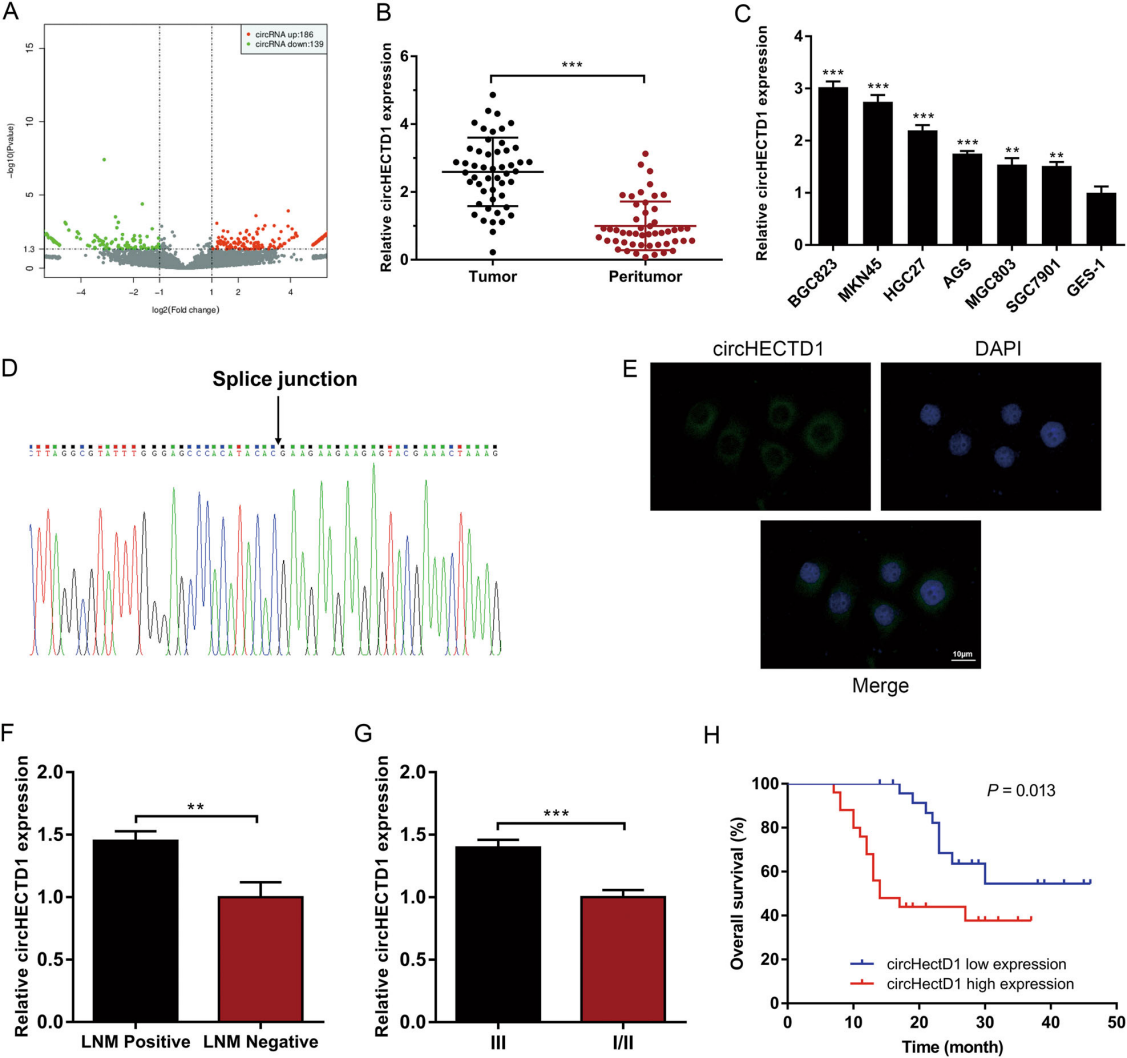
circHECTD1 is upregulated in gastric cancer (GC) and correlates with unfavorable prognosis. **a** Scatter plot showing differentially expressed circRNAs in GC tissues and paired peritumor samples. **b** circHECTD1 expression was examined in 50 pairs of GC tissues and peritumor samples using qRT-PCR. The expression level of circHECTD1 was significantly higher in GC tissues than in peritumor specimens. The data are shown as the mean ± SEM, *n* = 50. ***P < 0.001 vs. peritumor tissues. **c** circHECTD1 expression was investigated in GC cell lines (BGC823, MKN45, HGC27, AGS, MGC803, and SGC7901) and the normal human gastric mucosal epithelial cell line GES-1. The data are shown as the mean ± SEM, *n* = 3. **P < 0.01, ***P < 0.001 vs. GES-1. **d** Sanger sequencing was used to validate the presence of circHECTD1. The arrow indicates the head-to-tail splicing sites of circHECTD1. **e** Representative fluorescence in situ hybridization (FISH) images showing the dominantly cytoplasmic distribution of circHECTD1. **f** High circHECTD1 expression levels were observed in patients with lymph node metastasis (LNM). The data are shown as the mean ± SEM, *n* = 50. **P < 0.01 vs. the LNM negative group. **g** The expression level of circHECTD1 was higher in the AJCC stage III group than in the AJCC stage I/II group. The data are shown as the mean ± SEM, *n* = 50. ***P < 0.001 vs. the AJCC stage I/II group. **h** A Kaplan–Meier curve showed that the overall survival of GC patients with high circHECTD1 levels was worse than that of patients with low circHECTD1 levels. *P* = 0.013

Using ribosomal RNA-depleted total RNA acquired from three GC tissues and matched peritumor samples, we performed RNA sequencing and constructed a circRNA profile. The DESeq2 method was adopted to determine the differentially expressed circRNAs. As shown in Fig. 1a, 186 circRNAs were upregulated, and 139 circRNAs were downregulated in GC tissues (*P* < 0.05 and fold change >2.0). circHECTD1 was among the most significantly upregulated circRNAs in GC; thus, we chose circHECTD1 for further investigation. We then examined circHECTD1 expression levels in 50 paired GC tissues and peritumor specimens using quantitative real-time PCR (qRT-PCR). Consistent with the RNA sequencing results, the expression level of circHECTD1 was significantly higher in GC tissues than in matched peritumor samples (Fig. 1b). We then investigated the levels of circHECTD1 in GC cell lines and normal human gastric mucosal epithelial cell lines. As indicated in Fig. 1c, the expression levels of circHECTD1 were significantly higher in GC cell lines than in normal cell lines. Subsequently, we confirmed the head-to-tail splicing of the PCR product of circHECTD1 by Sanger sequencing (Fig. 1d). Fluorescence in situ hybridization (FISH) results indicated that circHECTD1 was predominantly located in the cytoplasm (Fig. 1e), which is consistent with previous reports^18,19^.

To assess the clinical significance of circHECTD1 in GC, we divided the patients into two groups based on the median value of circHECTD1 expression in GC tissues. As shown in Supplementary Table 1, the circHECTD1 expression level was associated with lymph node metastasis (LNM) (*P* = 0.038) and American Joint Committee on Cancer (AJCC) stage (*P* = 0.010). A higher level of circHECTD1 was observed in patients with LNM than in those without (Fig. 1f). Compared with the AJCC stage I/II group, patients with AJCC stage III had higher circHECTD1 expression (Fig. 1g). As demonstrated in Fig. 1h, a high circHECTD1 expression level was associated with poor overall survival (*P* = 0.013). Multivariate survival analysis indicated that high circHECTD1 levels independently predicted unfavorable survival outcomes in GC patients (Supplementary Table 2).

### circHECTD1 promotes GC cell proliferation, migration, and invasion

**Fig. 2:**
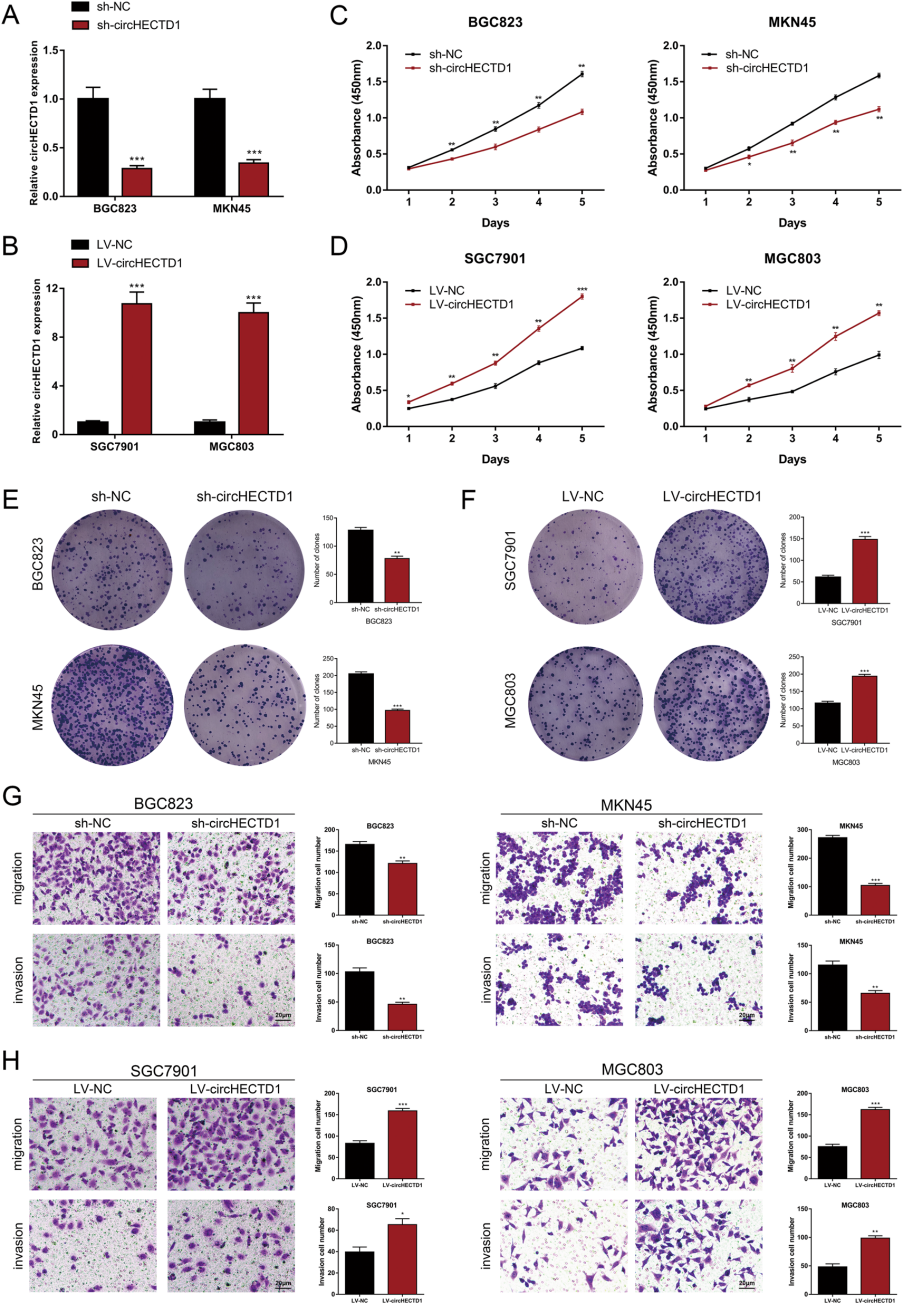
circHECTD1 promotes GC cell proliferation, migration, and invasion. **a** circHECTD1 was knocked down in BGC823 and MKN45 cells using shRNAs. **b** Lentiviruses were used to upregulate the expression of circHECTD1 in SGC7901 and MGC803 cells. **c** CCK-8 assays were performed to demonstrate cell proliferation in circHECTD1-silenced GC cells. **d** The proliferative ability was assessed in circHECTD1-overexpressing GC cells using CCK-8 assay. **e** Colony formation assays showed the clone numbers in GC cells with circHECTD1 knockdown. **f** Colony formation assays demonstrated the clone numbers in GC cells overexpressing circHECTD1. **g** Transwell assays were conducted to examine the effects of circHECTD1 knockdown on GC cell migration and invasion. **h** The effects of circHECTD1 upregulation on GC cell migration and invasion were examined using transwell assays. The data are shown as the mean ± SEM, *n* = 3. *P < 0.05, **P < 0.01, ***P < 0.001 vs. NC

To evaluate the functional roles of circHECTD1 in GC progression, we performed loss-of-function and gain-of-function assays. We first knocked down the expression of circHECTD1 in BGC823 and MKN45 cells using shRNAs (Fig. 2a). We also transfected SGC7901 and MGC803 cells with circHECTD1-overexpressing lentiviruses (Fig. 2b). Cell Count Kit-8 (CCK-8) assays revealed that circHECTD1 knockdown led to decreased proliferative ability in BGC823 and MKN45 cell lines (Fig. 2c). In contrast, overexpression of circHECTD1 promoted cell proliferation in SGC7901 and MGC803 cells (Fig. 2d). Colony formation assays further validated that circHECTD1 could facilitate GC cell proliferation (Fig. 2e, f). Moreover, transwell assays were conducted to assess the effect of circHECTD1 on GC cell motility. As demonstrated in Fig. 2g, we detected fewer migrated and invaded cells when using circHECTD1-silenced BGC823 and MKN45 cells. Conversely, GC cells overexpressing circHECTD1 had enhanced migration and invasion capacities (Fig. 2h). Collectively, these data showed that circHECTD1 facilitated the proliferation, migration, and invasion of GC cells.

### circHECTD1 enhances glutaminolysis in GC

**Fig. 3:**
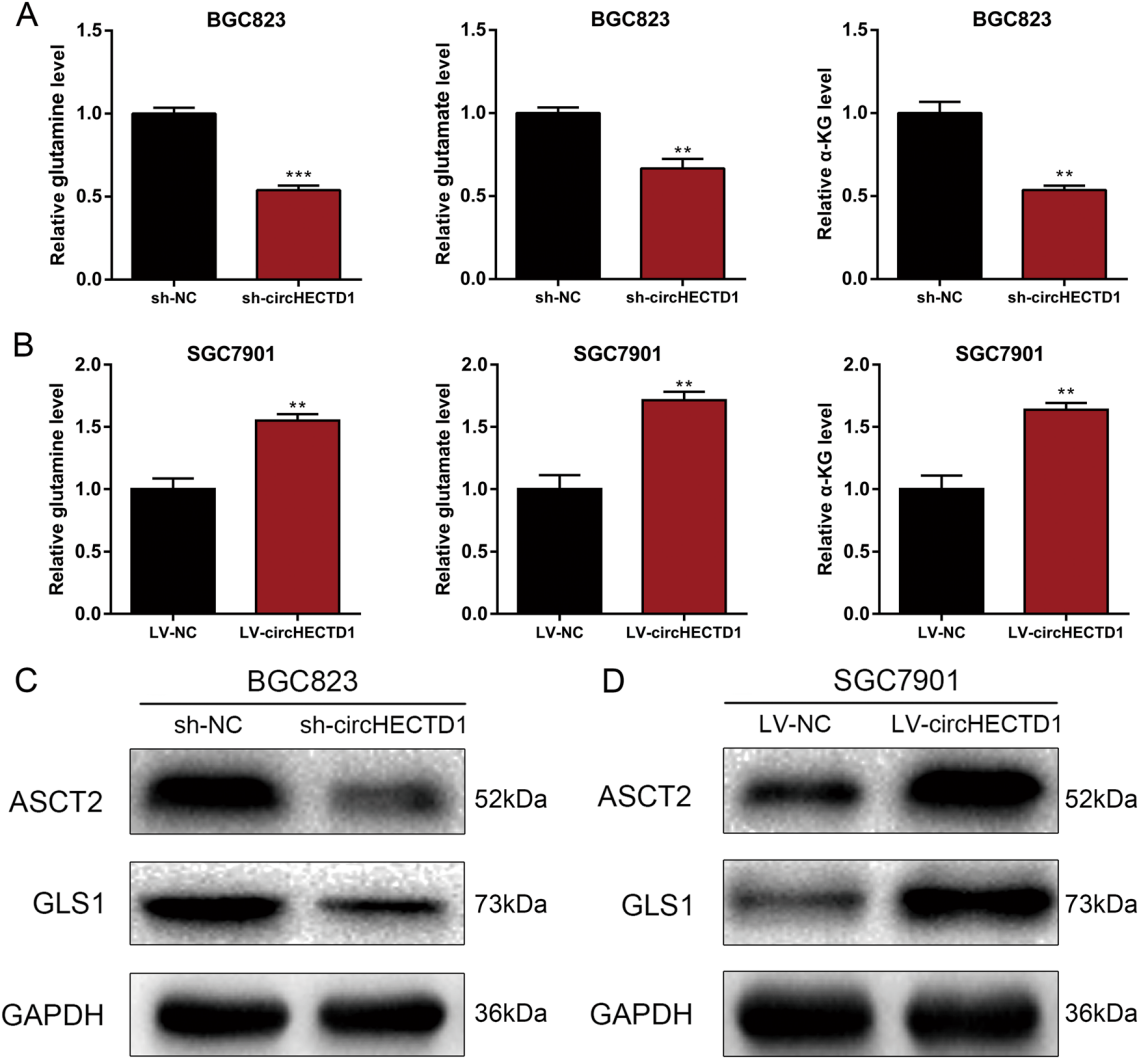
circHECTD1 enhances glutaminolysis in GC. **a** The levels of glutamine, glutamate, and α-KG were examined in circHECTD1-silenced BGC823 cells. **b** The levels of glutamine, glutamate, and α-KG were examined in circHECTD1-overexpressing SGC7901 cells. **c** Western blotting was performed to detect the expression levels of ASCT2 and GLS1 in circHECTD1-silenced BGC823 cells. **d** Western blotting was performed to detect the expression levels of ASCT2 and GLS1 in circHECTD1-overexpressing SGC7901 cells. The data are shown as the mean ± SEM, *n* = 3. **P < 0.01, ***P < 0.001 vs. NC

The effects of circHECTD1 on glutaminolysis were further explored. We analyzed the levels of glutaminolysis metabolites, and found that circHECTD1 knockdown resulted in decreased levels of glutamine, glutamate, and α-KG (Fig. 3a). Accordingly, overexpression of circHECTD1 increased glutamine, glutamate, and α-KG levels (Fig. 3b). Furthermore, we analyzed the expression levels of the glutamine importer ASCT2 and the glutaminolytic pathway-related rate-limiting enzyme GLS1. As indicated in Fig. 3c, d, we found that the protein levels of ASCT2 and GLS1 were increased in circHECTD1-overexpressing cells and decreased in circHECTD1-silenced GC cell lines. These results revealed the involvement of circHECTD1-mediated glutaminolysis in GC.

### circHECTD1 binds directly to miR-1256 to facilitate GC progression

**Fig. 4:**
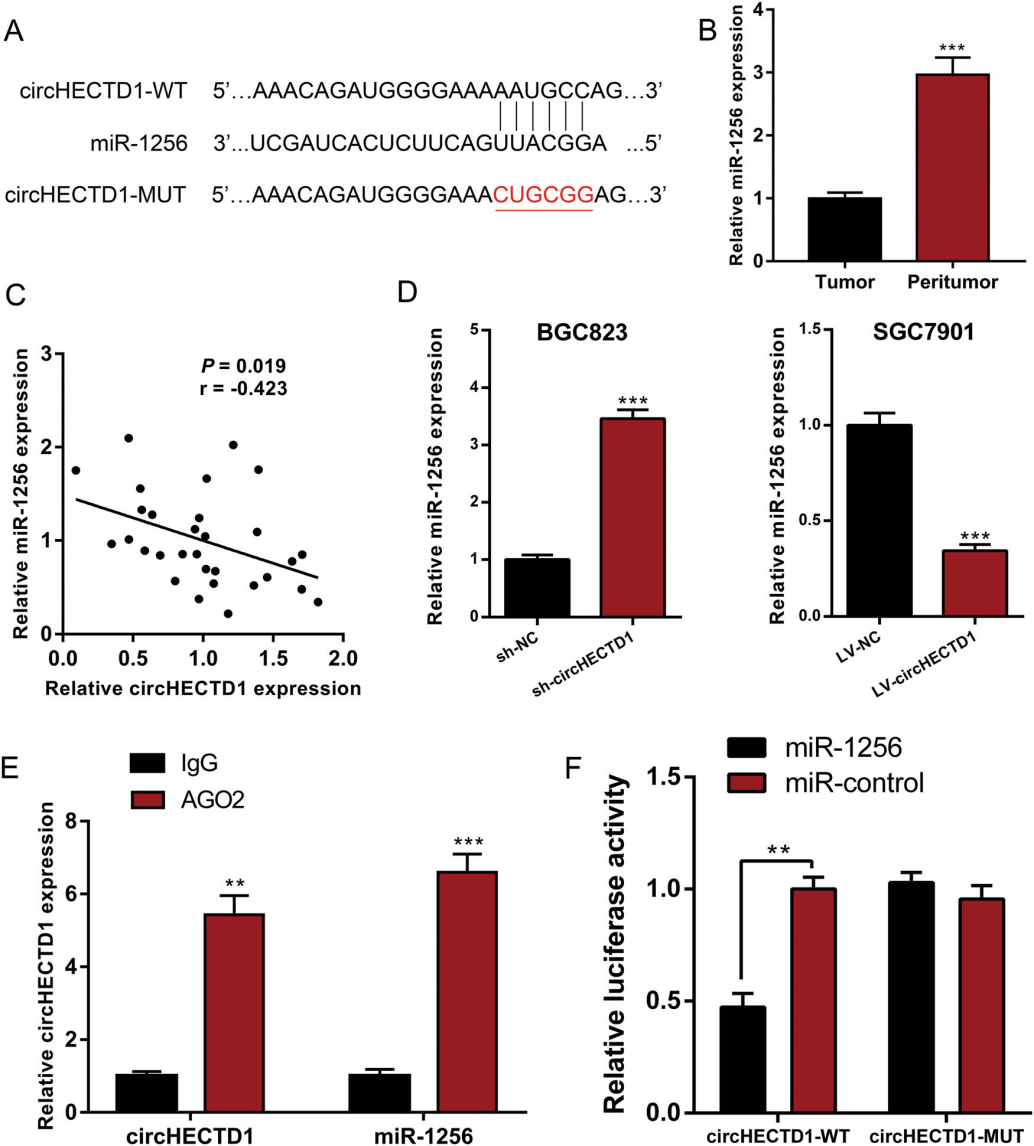
circHECTD1 binds directly to miR-1256 to facilitate GC progression. **a** Complementary sequence between circHECTD1 and miR-1256. **b** miR-1256 expression was examined in GC tissues and matched peritumor samples using qRT-PCR. The expression level of miR-1256 was significantly lower in GC tissues than in peritumor samples. The data are shown as the mean ± SEM, *n* = 30. ***P < 0.001 vs. peritumor tissues. **c** A significant negative correlation between circHECTD1 and miR-1256 was detected in GC tissues. *n* = 30, *P* = 0.019. **d** qRT-PCR was used to investigate the levels of miR-1256 in circHECTD1-silenced BGC823 cells and circHECTD1-overexpressing SGC7901 cells. The data are shown as the mean ± SEM, *n* = 3. ***P < 0.001 vs. NC. **e** An RNA immunoprecipitation (RIP) assay for AGO2 was performed to detect the levels of circHECTD1 and miR-1256 in AGO2 pellets. The data are shown as the mean ± SEM, *n* = 3. **P < 0.01, ***P < 0.001 vs. IgG. **f** Luciferase reporters containing wild-type (WT) or mutant (MUT) circHECTD1 transcripts were co-transfected with miR-1256 mimics or negative control. Luciferase activity was determined using the Dual-Luciferase Reporter System. The data are shown as the mean ± SEM, *n* = 3. **P < 0.01 vs. relative luciferase activity in the circHECTD1-WT with miR-control group

The cytoplasmic distribution of circHECTD1 suggested that it may target miRNA. We then used CircInteractome^20^ to predict the interacting miRNAs of circHECTD1 (Supplementary Table 3). Among the candidate miRNAs, miR-1256 was of particular interest (Fig. 4a). miR-1256 has been described as a tumor suppressor in prostate cancer^21^ and non-small cell lung cancer^22^. We examined the expression levels of miR-1256 in GC tissues and matched peritumor samples. qRT-PCR results showed that miR-1256 levels were dramatically decreased in GC tissues (Fig. 4b). As shown in Fig. 4c, a negative correlation between circHECTD1 expression and miR-1256 level was observed in GC tissues. We next assessed the levels of miR-1256 in GC cell lines with circHECTD1 knockdown or overexpression. Compared with control cells, BGC823 sh-circHECTD1 cells exhibited a higher miR-1256 level. In SGC7901 LV-circHECTD1 cells, the level of miR-1256 was markedly downregulated (Fig. 4d). Subsequently, we performed an RNA immunoprecipitation (RIP) assay for AGO2 and found higher levels of circHECTD1 and miR-1256 in the AGO2 pellet than in the control pellets (Fig. 4e). Moreover, a circHECTD1 fragment with wild-type (WT) or mutant (MUT) complementary binding sites was constructed and inserted into the luciferase reporter gene. The luc-circHECTD1-WT or luc-circHECTD1-MUT plasmid was cotransfected with miR-1256 mimics or negative control. The dual-luciferase reporter assay results revealed direct binding between circHECTD1 and miR-1256 (Fig. 4f).

**Fig. 5:**
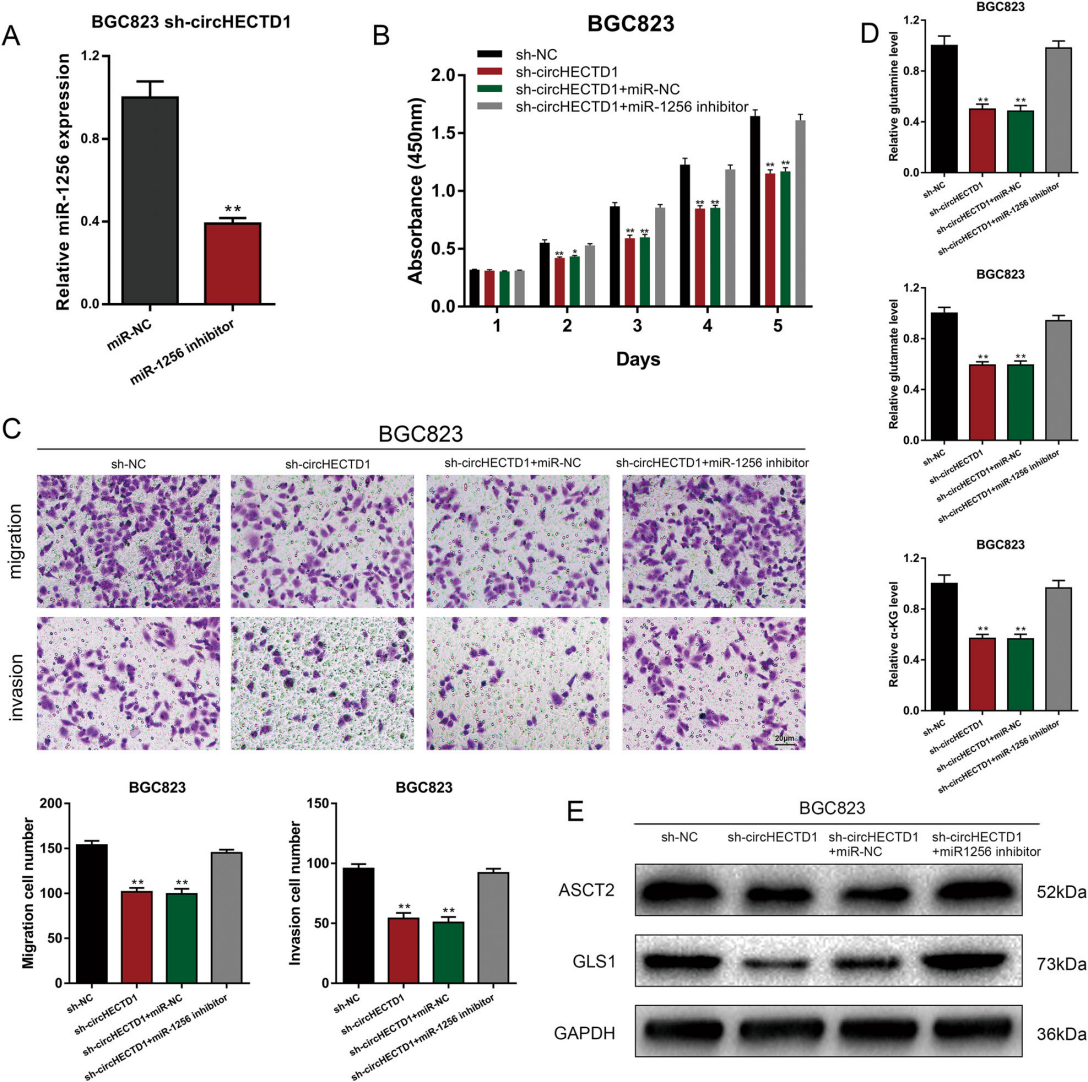
circHECTD1 enhances GC progression via miR-1256. **a** The efficiency of the miR-1256 inhibitor was verified using qRT-PCR. The data are shown as the mean ± SEM, *n* = 3. **P < 0.01 vs. miR-NC. **b** CCK-8 assays were performed to evaluate the effects of miR-1256 knockdown on the proliferative ability of BGC823 sh-circHECTD1 cells. The data are shown as the mean ± SEM, *n* = 3. *P < 0.05, **P < 0.01 vs. sh-NC. **c** Transwell assays were used to analyze the effects of miR-1256 knockdown on BGC823 sh-circHECTD1 cells. The data are shown as the mean ± SEM, *n* = 3. **P < 0.01 vs. sh-NC. **d** The levels of glutamine, glutamate, and α-KG were examined in BGC823 sh-circHECTD1 cells with miR-1256 knockdown. The data are shown as the mean ± SEM, *n* = 3. **P < 0.01 vs. sh-NC. **e** Western blotting was performed to detect the expression levels of ASCT2 and GLS1 in circHECTD1-silenced BGC823 cells with miR-1256 knockdown

To demonstrate that circHECTD1 enhanced GC progression and glutaminolysis through miR-1256, we downregulated the expression of miR-1256 in BGC823 sh-circHECTD1 cells (Fig. 5a) and performed a series of rescue experiments. CCK-8 and transwell assays showed that inhibition of miR-1256 could reverse the inhibitory effects of circHECTD1 knockdown on GC cell proliferation and motility (Fig. 5b, c). We then detected the levels of glutamine, glutamate, and α-KG in BGC823 sh-circHECTD1 cells treated with a miR-1256 inhibitor. The results demonstrated that suppression of miR-1256 could upregulate the decreased levels of glutaminolysis metabolites in BGC823 sh-circHECTD1 cells (Fig. 5d). Accordingly, ASCT2 and GLS1 levels in BGC823 sh-circHECTD1 cells were restored after miR-1256 treatment (Fig. 5e). Collectively, the results suggested the tumor-promoting role of the circHECTD1/miR-1256 axis in GC.

### USP5 is downstream of the circHECTD1/miR-1256 axis

**Fig. 6:**
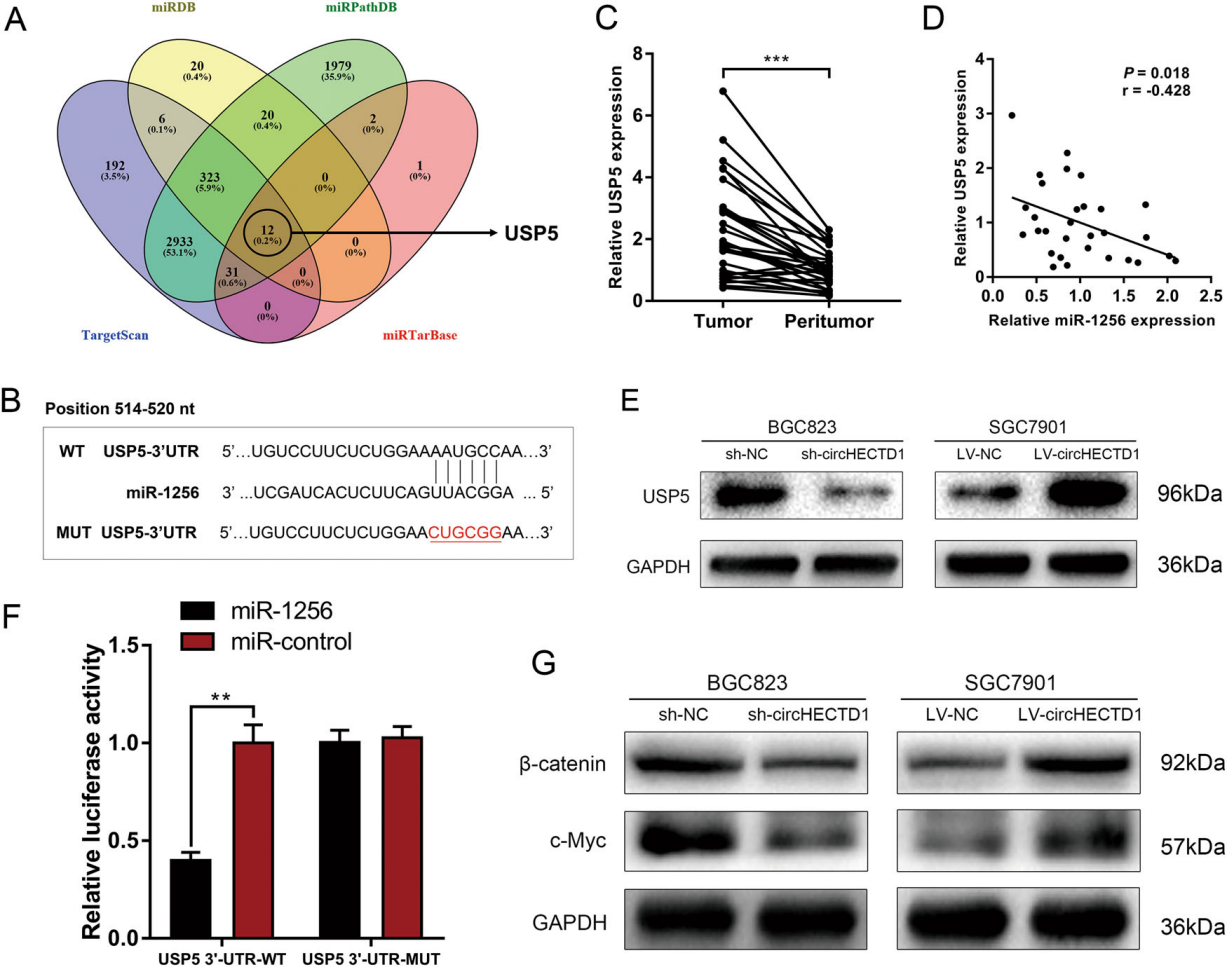
USP5 is downstream of the circHECTD1/miR-1256 axis. **a** Bioinformatics tools (TargetScan, miRDB, miRPathDB, and miRTarBase) were used to predict the downstream targets of miR-1256. USP5 was predicted by all four algorithms. **b** Complementary sequence between USP5 and miR-1256. **c** The expression levels of USP5 in GC tissues and matched peritumor samples were examined by qRT-PCR. The data are shown as the mean ± SEM, *n* = 30. ***P < 0.001 vs. peritumor tissues. **d** A reverse correlation between miR-1256 and USP5 was detected in GC tissues. *n* = 30, *P* = 0.018. **e** Western blotting was used to investigate the levels of USP5 in circHECTD1-silenced BGC823 cells and circHECTD1-overexpressing SGC7901 cells. **f** A dual-luciferase reporter assay was conducted to confirm that USP5 was a downstream target of miR-1256. The data are shown as the mean ± SEM, *n* = 3. **P < 0.01 vs. relative luciferase activity in the USP5 3′-UTR WT with miR-control group. **g** The expression levels of β-catenin and c-Myc were examined by Western blotting in GC cells with circHECTD1 knockdown or overexpression

To understand the downstream effectors of the circHECTD1/miR-1256 pathway, we combined four bioinformatics tools (TargetScan, miRDB, miRPathDB, and miRTarBase) to predict the target genes of miR-1256. Among the 12 genes predicted by all four prediction algorithms, USP5 was of particular interest (Fig. 6a). The complementary sequence between USP5 and miR-1256 is shown in Fig. 6b. USP5 has been reported to be upregulated in several cancers and can activate the β-catenin/c-Myc signaling pathway^23,24^, which is an important glutaminolytic-associated pathway. We evaluated the expression levels of USP5 in GC using qRT-PCR and found that USP5 was significantly higher in GC tissues than in peritumor specimens (Fig. 6c). Further analysis showed that miR-1256 was negatively correlated with USP5 expression level (Fig. 6d). In addition, the level of USP5 was higher in GC cells with miR-1256 inhibition, whereas a lower expression level of USP5 was detected in BGC823 cells treated with miR-1256 mimics (Fig. 6e). A dual-luciferase reporter assay confirmed that USP5 was a downstream target of miR-1256 (Fig. 6f). Western blotting assays indicated that the expression levels of β-catenin and c-Myc were inhibited after circHECTD1 knockdown, while the β-catenin/c-Myc pathway was activated upon circHECTD1 overexpression (Fig. 6g).

**Fig. 7:**
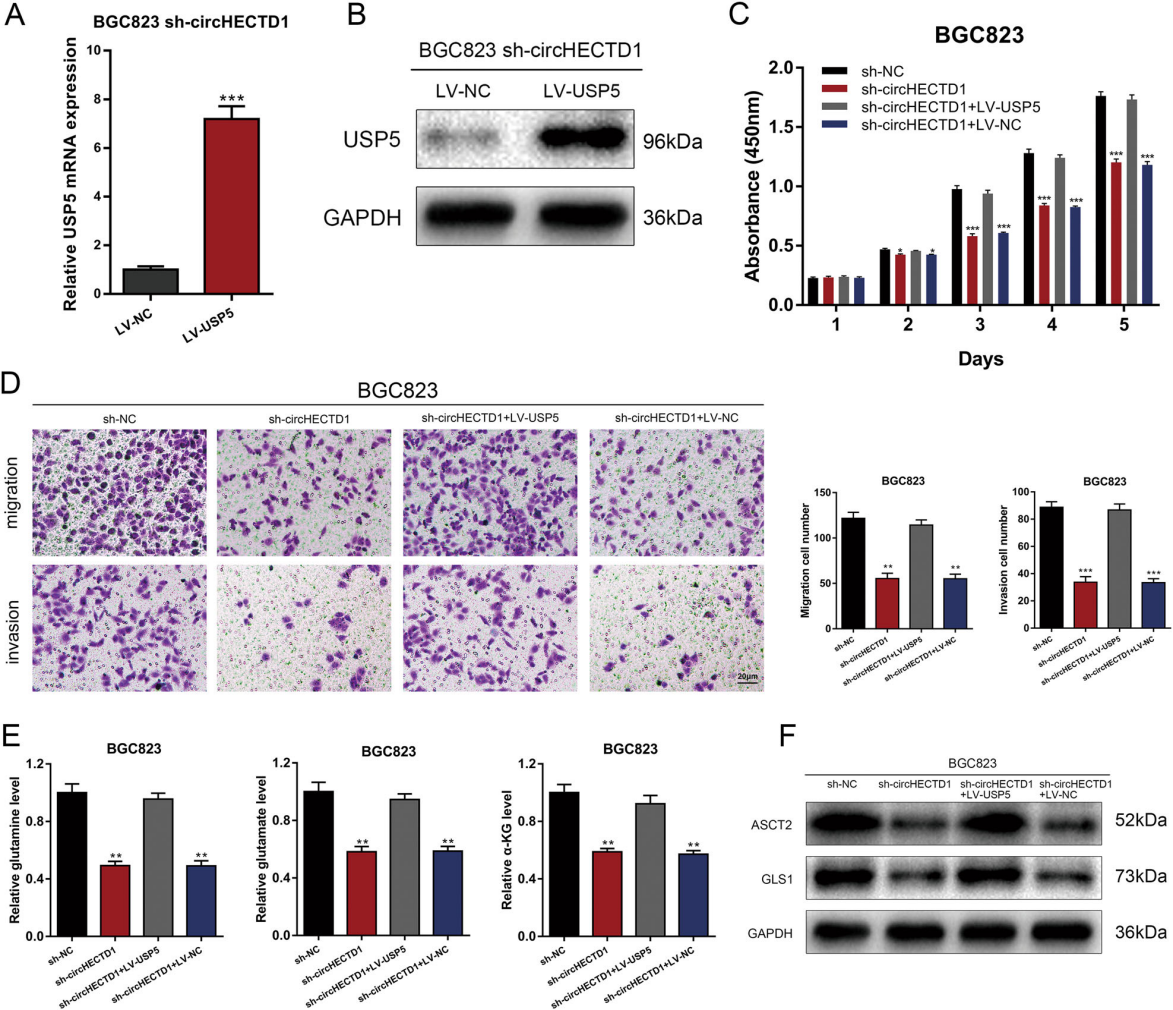
The circHECTD1/miR-1256 axis affected GC progression through USP5. **a** The efficiency of USP5 overexpression was verified by qRT-PCR. The data are shown as the mean ± SEM, *n* = 3. ***P < 0.001 vs. LV-NC. **b** The efficiency of USP5 overexpression was verified by Western blotting. **c** CCK-8 assays were performed to evaluate the effects of USP5 overexpression on the proliferative ability of BGC823 sh-circHECTD1 cells. The data are shown as the mean ± SEM, *n* = 3. *P < 0.05, ***P < 0.001 vs. sh-NC. **d** Transwell assays were used to analyze the effects of USP5 overexpression on BGC823 sh-circHECTD1 cells. The data are shown as the mean ± SEM, *n* = 3. **P < 0.01, ***P < 0.001 vs. sh-NC. **e** The levels of glutamine, glutamate, and α-KG were examined in BGC823 sh-circHECTD1 cells overexpressing USP5. The data are shown as the mean ± SEM, *n* = 3. **P < 0.01 vs. sh-NC. **f** Western blotting was performed to detect the expression levels o**f** ASCT2 and GLS1 in circHECTD1-silenced BGC823 cells overexpressing USP5

Rescue experiments were performed to confirm that the circHECTD1/miR-1256 pathway affected GC progression and glutaminolysis via USP5. We next overexpressed USP5 in circHECTD1-silenced BGC823 cells (Fig. 7a, b). CCK-8 and transwell assays showed that the inhibited proliferation and motility capacities in BGC823 sh-circHECTD1 cells were increased after USP5 overexpression (Fig. 7c, d). Levels of glutaminolysis metabolites, ASCT2, and GLS1 were also rescued after overexpressing USP5 in circHECTD1-silenced GC cells (Fig. 7e, f). Consequently, we suggest that the circHECTD1/miR-1256/USP5 axis is involved in GC progression and glutaminolysis.

### circHECTD1 exerted tumor-promoting effects in GC in vivo

**Fig. 8:**
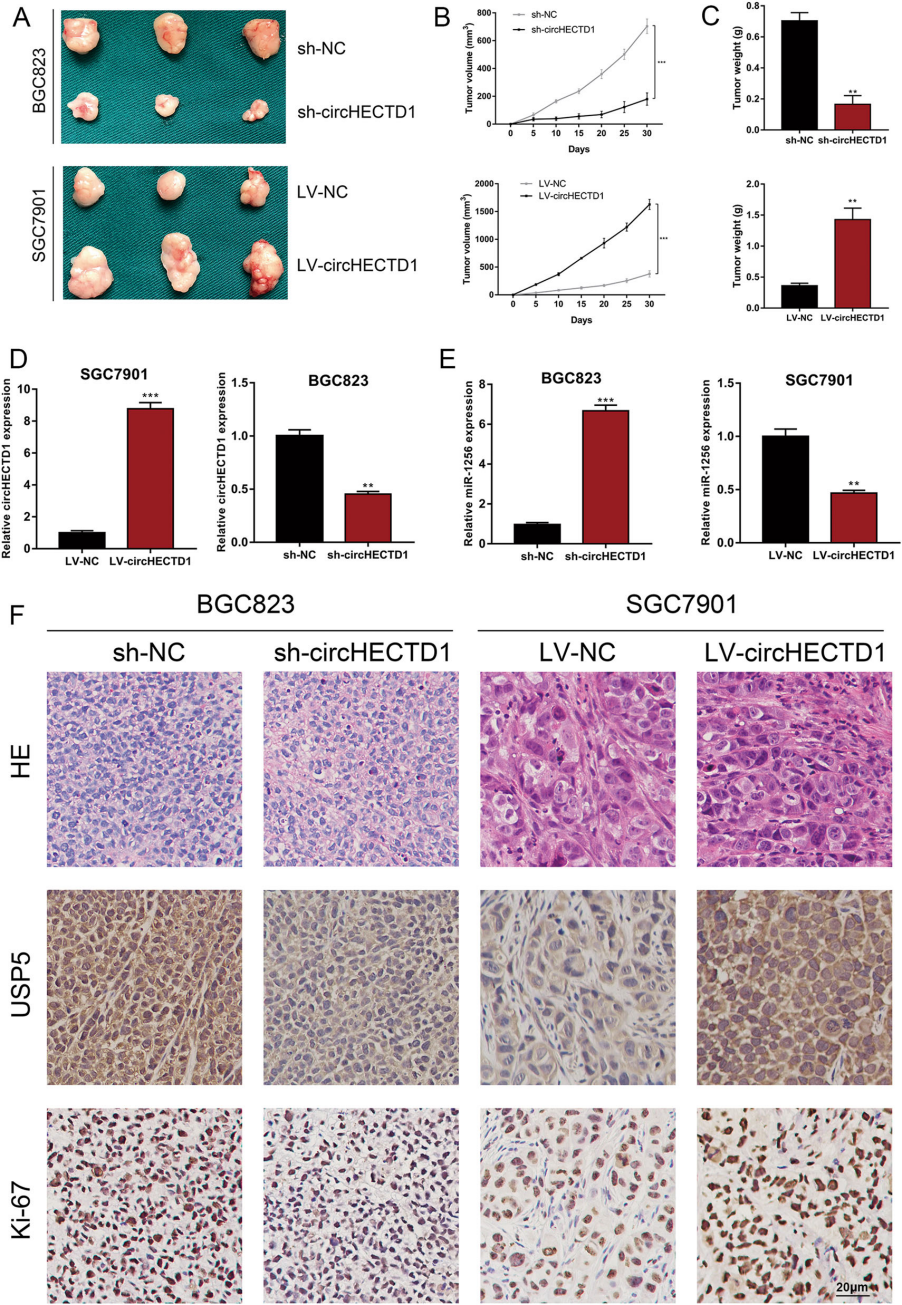
circHECTD1 exerted tumor-promoting effects in GC in vivo. **a** Xenograft mouse models were used to assess the effects of circHECTD1 on GC growth. Nude mice were injected subcutaneously with GC cells with circHECTD1 knockdown or overexpression and corresponding control cells. The xenografts were harvested after 1 month. **b** The volumes of the xenografts were measured at the indicated time points. The data are shown as the mean ± SEM, *n* = 3. ***P < 0.001 vs. NC. **c** The xenograft weights were compared between different groups. The data are shown as the mean ± SEM, *n* = 3. ***P < 0.001 vs. NC. **d** qRT-PCR was performed to detect the expression level of circHECTD1 in the xenografts. The data are shown as the mean ± SEM, *n* = 3. **P < 0.01, ***P < 0.001 vs. NC. **e** The expression level of miR-1256 was assessed in the xenografts. The data are shown as the mean ± SEM, *n* = 3. **P < 0.01, ***P < 0.001 vs. NC. **f** Representative Ki-67 and USP5 immunostaining of the xenografts

**Fig. 9:**
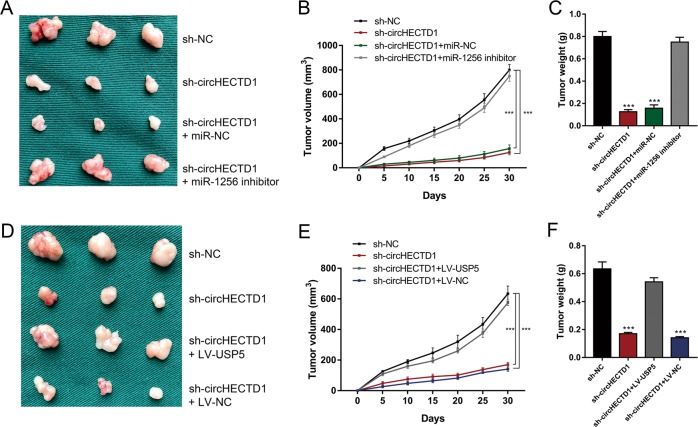
circHECTD1 facilitated GC progression in vivo by targeting miR-1256, thus upregulating USP5 expression. **a** Xenograft tumors in nude mice from the four treatment groups (sh-NC, sh-circHECTD1, sh-circHECTD1 + miR-NC, sh-circHECTD1 + miR-1256 inhibitor) after subcutaneous injection of BGC823 cells (*n* = 3 for each group). **b** Xenograft volumes from the four treatment groups (sh-NC, sh-circHECTD1, sh-circHECTD1 + miR-NC, sh-circHECTD1 + miR-1256 inhibitor) were measured at the indicated time points. The data are shown as the mean ± SEM, *n* = 3. ***P < 0.001 vs. sh-NC. **c** Xenograft weights were compared between different groups (sh-NC, sh-circHECTD1, sh-circHECTD1 + miR-NC, sh-circHECTD1 + miR-1256 inhibitor). The data are shown as the mean ± SEM, *n* = 3. ***P < 0.001 vs. sh-NC. **d** Xenograft tumors in nude mice from the four treatment groups (sh-NC, sh-circHECTD1, sh-circHECTD1 + LV-USP5, sh-circHECTD1 + LV-NC) after subcutaneous injection of BGC823 cells (*n* = 3 for each group). **e** Xenograft volumes from the four treatment groups (sh-NC, sh-circHECTD1, sh-circHECTD1 + LV-USP5, sh-circHECTD1 + LV-NC) were measured at the indicated time points. The data are shown as the mean ± SEM, *n* = 3. ***P < 0.001 vs. sh-NC. **f** Xenograft weights were compared between different groups (sh-NC, sh-circHECTD1, sh-circHECTD1 + LV-USP5, sh-circHECTD1 + LV-NC). The data are shown as the mean ± SEM, *n* = 3. ***P < 0.001 vs. sh-NC

We utilized in vivo xenograft mouse models to assess the effects of circHECTD1 on GC growth in vivo. We inoculated nude mice with circHECTD1-silenced or circHECTD1-overexpressing GC cells and the corresponding control cells. After 1 month, the xenografts were harvested. As demonstrated in Fig. 8a, smaller tumor sizes were detected in mice injected with circHECTD1-silenced GC cells, and larger xenograft sizes were observed in mice inoculated with circHECTD1-overexpressing cells. The xenografts grew slower in the BGC823 sh-circHECTD1 group, while the tumors grew faster in the SGC7901 LV-circHECTD1 group (Fig. 8b). The weights of the xenografts confirmed the tumor-promoting effects of circHECTD1 in vivo (Fig. 8c). We also performed qRT-PCR to validate the consistent knockdown or overexpression of circHECTD1 in the xenografts (Fig. 8d). The expression of miR-1256 was higher in the xenografts from the BGC823 sh-circHECTD1 group, and lower miR-1256 levels were observed in the tumors from the SGC7901 LV-circHECTD1 group (Fig. 8e). Immunohistochemistry (IHC) results showed that Ki-67 and USP5 expression levels were decreased in the BGC823 sh-circHECTD1 group and increased in the SGC7901 LV-circHECTD1 group (Fig. 8f). To further validate the effects of the circHECTD1/miR-1256/USP5 axis, we performed rescue experiments in vivo. As shown in Fig. 9a–c, suppressed xenograft growth in the BGC823 sh-circHECTD1 group was counteracted by miR-1256 inhibition. In addition, USP5 overexpression in BGC823 sh-circHECTD1 cells significantly reversed the inhibitory effects of circHECTD1 knockdown on tumor volume and weight (Fig. 9d–f).

## Discussion

An increasing number of studies, especially in the field of cancer, have shown that noncoding RNAs play vital regulatory roles in multiple physiological and pathological processes^25,26^. Mounting evidence shows the involvement of circRNAs in human malignancies^27,28^, and circHECTD1 has been recently reported to be involved in silicosis and stroke. circHECTD1 promotes endothelial-mesenchymal transition and macrophage activation after SiO2 exposure by competing with the pre-mRNA of its host gene hectd1^19,29^. In cerebral ischemic stroke, circHECTD1 inhibits TIPARP expression, and subsequently suppresses astrocyte activation by targeting miR-142^18^. However, its role in cancer has never been investigated. In the present study, we discovered that circHECTD1 was upregulated in GC tissues and cell lines. High circHECTD1 levels were associated with unfavorable clinicopathological features and poor overall survival. Functional experiments revealed that circHECTD1 facilitated GC cell progression in vitro and in vivo.

Glutaminolysis has been acknowledged as an indispensable metabolic process that supports cancer progression^30,31^. The involvement of miRNAs and long non-coding RNAs in glutaminolysis has been established in tumor progression^32,33^. However, the role of circRNAs in glutamine metabolic reprogramming remains unclear. Here, for the first time, we correlated glutaminolysis with dysregulated circRNAs in GC. Knockdown of circHECTD1 decreased the levels of glutamine, glutamate, and α-KG, while circHECTD1 overexpression increased the levels of these glutaminolysis metabolites. Further investigation showed that circHECTD1 modulated the expression levels of ASCT2 and GLS1, which are two crucial enzymes involved in the process of glutamine metabolism.

Given that circHECTD1 is located predominantly in the cytoplasm, we speculated that circHECTD1 might directly target certain miRNAs in GC. The results indicated that circHECTD1 targeted miR-1256 to exert its tumor-promoting effects. miR-1256 has been reported as a tumor suppressor in several types of cancers. Colorectal cancer patients with lower miR-1256 levels exhibit worse survival outcomes, and the miR-1256 level is an independent predictor of patient prognosis^34^. In non-small cell lung cancer, miR-1256 targets TCNC1 to inhibit cell proliferation and migration^22^. Decreased miR-1256 expression is detected in nasopharyngeal carcinoma and is associated with dysregulated JNK2 levels^35^. miR-1256 directly targets TRIM68 and contributes to the suppression of prostate cancer progression^21^. In GC, we found that the miR-1256 expression level was decreased compared with that in peritumor samples. As shown by RIP and dual-luciferase reporter assays, circHECTD1 could bind to and interact with miR-1256. Through targeting miR-1256, circHECTD1 inhibited the proliferation, motility, and glutaminolysis of GC cells.

To further elucidate the mechanisms underlying circHECTD1/miR-1256-mediated GC progression, we explored the downstream effectors. Bioinformatics prediction suggested that USP5, a member of the deubiquitinase family, was a downstream target of miR-1256. USP5 contributes to the carcinogenesis of multiple tumors, including lung cancer^23^, pancreatic cancer^36^, multiple myeloma^37^, liver cancer^38^, and glioblastoma^39^. In the present study, we demonstrated that the expression level of USP5 was increased in GC and that USP5 is a downstream target of the circHECTD1/miR-1256 axis. In addition, previous studies showed that USP5 is involved in β-catenin stabilization^23,24^. In non-small cell lung cancer, USP5 deubiquitinates β-catenin and leads to the activation of the β-catenin/c-Myc signaling pathway^23^. USP5 can also activate β-catenin/c-Myc signaling via stabilizing FoxM1^24^. The β-catenin/c-Myc signaling pathway is regarded as one of the crucial regulatory pathways responsible for glutaminolysis and cancer progression. In tumor cells, β-catenin signaling is activated and consequently provokes c-Myc-mediated glutaminolysis^40^. On the one hand, c-Myc can directly increase the expression levels of the surface transporters SN2 and SLC1A5. On the other hand, c-Myc can indirectly activate GLS1 via suppressing miR-23a/b^41,42^. Thus, the activation of β-catenin/c-Myc can drive glutaminolysis. Liu et al. reported that gankyrin leads to enhanced glutaminolysis by upregulating β-catenin/c-Myc signaling in hepatocellular carcinoma^6^. Our data revealed that the circHECTD1/miR-1256/USP5 pathway regulated GC progression by modulating β-catenin expression and downstream c-Myc levels.

## Conclusion

In summary, the current study showed that the expression of circHECTD1 was increased in GC tissues and associated with overall survival. circHECTD1 facilitated GC cell glutaminolysis, growth, and invasiveness. Mechanistic studies revealed that circHECTD1 targeted miR-1256 to regulate USP5 expression levels and modulate the downstream β-catenin/c-Myc signaling pathway. circHECTD1 is a glutaminolysis-associated circRNA that could serve as a potential metabolic drug target for GC treatment.

## Materials and methods

### Clinical specimens and ethical statement

GC and paired peritumor tissues were acquired from GC patients who underwent radical gastrectomy at the First Affiliated Hospital of Wannan Medical College. None of the enrolled patients had received preoperative radiotherapy or chemotherapy. Tissue samples were immediately stored in liquid nitrogen after surgery until use. Written informed consent was obtained from all patients before operation. This study was approved by the Ethics Committee of the First Affiliated Hospital of Wannan Medical College.

### Library construction and RNA sequencing

Total RNA was extracted from tissues and cells using TRIzol reagent (Invitrogen, Carlsbad, CA, USA). An ND-1000 Nanodrop was used to evaluate RNA purity, and an Agilent 2200 TapeStation (Agilent Technologies, Santa Clara, CA, USA) was adopted to assess RNA integrity. An Epicentre Ribo-Zero rRNA Removal Kit (Illumina, San Diego, CA, USA) was used to remove rRNA from total RNA. RNase R (Epicentre Technologies, Madison, WI, USA) was adopted to degrade linear RNA. Following adaptor ligation and enrichment with a low cycle based on the instructions of the NEBNext® Ultra™ RNA Library Prep Kit for Illumina, purified RNA fragments were used to synthesize cDNA and subsequently sequenced on a HiSeq 3000 in 2 × 150 bp mode. After pre-processing the sequencing reads and quality control, we employed CIRI2 and CIRCexplorer2 to detect circRNAs. The DESeq2 method was used to determine the differentially expressed circRNAs (*P* < 0.05 and fold change >2.0).

### Real-time PCR (qRT-PCR)

cDNA was synthesized using PrimeScript RT Master Mix (TaKaRa, Dalian, China). qRT-PCR was performed with TB Green Premix Ex Taq (TaKaRa) according to the manufacturer’s instructions. The relative expression of RNAs was calculated by the 2^−ΔΔCt^ quantification method. qRT-PCR amplification was performed using the following primers: circHECTD1: forward: 5′-ACTCCGTCACCTCGATTAGC-3′; reverse: 5′-ATCATCCCATGTTCTCCGGC-3′; USP5: forward: 5′-CGGATTTGACCTTAGCG-3′; reverse: 5′-CTGCCATCGAAGTAGCG-3′; GAPDH: forward: 5′-AATCCCATCACCATCTTCC-3′; reverse: 5′-CATCACGCCACAGTTTCC-3′. The qRT-PCR primers for miR-1256 and U6 were designed and synthesized by RiboBio (Guangzhou, China).

### Cell lines and culture

The human GC cell lines BGC823, MKN45, HGC27, AGS, MGC803, and SGC7901 and the normal human gastric mucosal epithelial cell line GES-1 were purchased from the Shanghai Institute of Cell Biology, Chinese Academy of Sciences (Shanghai, China). All cell lines were cultured in RPMI-1640 (Gibco, Carlsbad, CA, USA) containing 10% fetal bovine serum (FBS) supplemented with 100 U/mL penicillin and 100 μg/mL streptomycin (HyClone, Logan, UT, USA) in a humidified atmosphere at 37 °C with 5% CO_2_.

### Fluorescence in situ hybridization (FISH)

Hybridization was performed overnight with a circHECTD1 FISH probe. Nuclei were stained with DAPI. Images were obtained using a confocal microscope (Leica Microsystems, Mannheim, Germany). Detailed procedures were described previously^18^.

### Lentivirus transfection, miRNA mimics, and inhibitors

Lentiviral-based shRNA targeting circHECTD1 and lentiviruses overexpressing circHECTD1 or USP5 were purchased from GeneChem (Shanghai, China). The transfection efficiency was confirmed using qRT-PCR or Western blotting. Puromycin (1 μg/mL) was used to select stable cells for 2 weeks. miR-1256 mimics and inhibitors were purchased from RiboBio (Guangzhou, China).

### Cell proliferation assays

For the CCK-8 assay, GC cells were seeded into 96-well plates (1 × 10^3^ cells/well) and cultured at 37 °C with 5% CO_2_ for 1–5 days. At the indicated time points, 10 μL of CCK-8 reagent (Dojindo Laboratories, Kumamoto, Japan) was added to the culture medium. After incubation for 2 h, the absorbance of each well was recorded at a wavelength of 450 nm using a Biotek ELx800 plate reader (BioTek Instruments, Inc., Winooski, VT, USA). For the colony formation assay, GC cells were seeded into six-well plates at a density of 500 cells/well, and incubated at 37 °C in a humidified atmosphere of 5% CO_2_ for 2 weeks. The colonies were then fixed with 4% paraformaldehyde and stained with a 0.1% crystal violet solution. The colonies were photographed and counted.

### Transwell assays

For the transwell migration assay, 2 × 10^4^ cells were suspended in 250 μL of serum-free culture medium and added to the upper chamber. For the invasion assay, the transwell chamber was precoated with Matrigel (BD Biosciences, San Jose, CA, USA), and the same number of cells was added to the upper chamber. Next, 500 μL of RPMI-1640 containing 10% FBS was added to the lower chamber. The cells in the upper chamber were removed after incubation for 24 h, and the cells that migrated or invaded to the lower membrane surface were fixed with 4% paraformaldehyde and stained with a 0.1% crystal violet solution. The migrated or invaded cells were then photographed and counted.

### Measurement of glutamine, glutamate, and α-KG levels

Concentrations of glutamine and glutamate were assessed using a Glutamine/Glutamate Determination Kit (Sigma-Aldrich, St. Louis, MO, USA) according to the manufacturer’s instructions. The α-KG level was measured using an α-KG Assay Kit (Abcam, Cambridge, MA, USA) according to the manufacturer’s protocols.

### Western blotting assay

GC tissues and cells were lysed in ice-cold RIPA buffer with 1 mM PMSF. Total protein was separated by SDS-PAGE, transferred to PVDF membranes (Millipore, Bedford, MA, USA) and blocked with 5% skim milk in TBST for 2 h. Then, the membranes were incubated with primary antibodies specific for ASCT2, GLS1, USP5, β-catenin, c-Myc and GAPDH at 4 °C overnight, followed by incubation with horseradish peroxidase-conjugated secondary antibodies (Cell Signaling Technology, Danvers, MA, USA) at room temperature for 2 h. The signals were visualized with Super ECL Detection Reagent (Yeasen, Shanghai, China), and images were acquired using Image Lab software (Bio-Rad, Hercules, CA, USA).

### RIP assay

RIP assays were performed using a Magna RIP RNA-Binding Protein Immunoprecipitation Kit (Millipore, Billerica, MA, USA) according to the manufacturer’s protocol. Briefly, cells were lysed using RIP lysis buffer containing protease and RNase inhibitors (Millipore). Then, the cell lysates were incubated with an anti-AGO2 antibody or nonspecific IgG antibody (Abcam) at 4 °C overnight. The immunoprecipitated RNAs were eluted with proteinase K, and circHECTD1 enrichment was detected by qRT-PCR.

### Dual-luciferase reporter assay

Dual-luciferase reporter assays were performed as previously described^43^. Cells (5 × 10^4^ cells/well) were seeded into 24-well plates. After cotransfection with the constructed luciferase plasmids and miR-1256 mimics or negative control using Lipofectamine 3000 reagent (Invitrogen) for 48 h, firefly and renilla luciferase activities were measured using the Dual-Luciferase Reporter Assay System (Promega, Madison, WI, USA) following the manufacturer’s protocol.

### Animal experiments

Xenograft mouse models were constructed to examine the effects of circHECTD1 on GC tumor growth in vivo. GC cells with circHECTD1 overexpression or knockdown and corresponding control cells were injected subcutaneously into the flanks of 4-week-old BALB/c nude mice (5 × 10^6^ cells resuspended in 200 mL of culture medium). Tumors were measured routinely with a caliper. The xenograft volume was calculated as (length × width^2^)/2. After 1 month, the xenografts were harvested and weighed. The xenograft tissues were then used for qRT-PCR and immunohistochemical analysis. All animal experiments were approved by the Animal Ethics Committee of the First Affiliated Hospital of Wannan Medical College.

### IHC examination

Tissue samples were fixed in 4% paraformaldehyde, embedded in paraffin, and sectioned. The tissue sections were incubated with anti-USP5 and anti-Ki-67 primary antibodies at 4 °C overnight and then incubated with an HRP-conjugated secondary antibody.

### Statistical analysis

The results are presented as the mean ± SEM. All statistical analyses were performed using GraphPad Prism 8.0 and SPSS 22.0. Differences between two groups were compared using two-tailed Student’s *t* test. Fisher’s exact test or chi-square test was performed to examine the relationship between circHECTD1 levels and clinicopathological features. Survival analysis was performed according to the Kaplan–Meier method and Cox proportional hazards model. The correlation analysis was conducted using the Pearson correlation coefficient. *P* < 0.05 was considered to be statistically significant.

## Supplementary information

Supplementary tables
