# Supplementary material for: circHECTD1 facilitates glutaminolysis to promote gastric cancer progression by targeting miR-1256 and activating β-catenin/c-Myc signaling
Source: Cell Death Dis. 2019 Aug 2;10(8):576. doi: 10.1038/s41419-019-1814-8 (PMC6675787; doi:10.1038/s41419-019-1814-8)
Supplement: Supplementary file 1 — Supplementary tables [file 41419_2019_1814_MOESM1_ESM.docx]

**Supplementary Table 1 Correlation between circHECTD1 expression and clinicopathological features of GC patients (*n* = 50)**

| **Clinicopathological features** | **Low circHECTD1 expression (*n* = 25)** | **High circHECTD1 expression (*n* = 25)** | ***P*** |
| --- | --- | --- | --- |
| Age  < 60 years  ≥ 60 years | 13  12 | 7  18 | 0.148 |
| Gender  Female  Male | 6  19 | 10  15 | 0.364 |
| Tumor location |  |  | 0.776 |
| Down | 15 | 13 |  |
| Upper/Middle | 10 | 12 |  |
| Tumor size |  |  | 0.088 |
| < 5 cm | 15 | 8 |  |
| ≥ 5 cm | 10 | 17 |  |
| Differentiation  Well/Moderate | 12 | 13 | 1.000 |
| Poor | 13 | 12 |  |
| Vascular invasion  No  Yes | 11  14 | 7  18 | 0.377 |
| Lymph node metastasis  Negative  Positive | 13  12 | 5  20 | 0.038* |
| AJCC stage  I/II  III | 16  9 | 6  19 | 0.010* |

*AJCC* American Joint Committee on Cancer

* *P* < 0.05

| **Variables** | **Overall survival** | | | | |
| --- | --- | --- | --- | --- | --- |
|  | **Univariate Multivariate** | | | | |
|  | **Log-rank** | ***P*** | **HR** | **95% CI** | ***P*** |
| Age (≥60 years vs <60 years) | 0.008 | 0.927 |  |  |  |
| Gender (Male vs Female) | 1.732 | 0.188 |  |  |  |
| Tumor location (Upper/Middle vs Down) | 0.342 | 0.559 |  |  |  |
| Tumor size (≥ 5 cm vs < 5cm) | 0.083 | 0.773 |  |  |  |
| Differentiation (Poor vs Well/Moderate) | 0.957 | 0.328 |  |  |  |
| Vascular invasion (Yes vs No) | 4.757 | 0.029* | Not included |  |  |
| Lymph node metastasis (Positive vs Negative) | 4.663 | 0.031* | 2.616 | 1.022-6.699 | 0.045* |
| AJCC stage (III vs I/II) | 11.650 | 0.001* | 3.501 | 1.336-9.173 | 0.011* |
| circHECTD1 expression (High vs Low) | 6.202 | 0.013* | 3.332 | 1.340-8.285 | 0.010* |

**Supplementary Table 2** **Univariate and multivariable analysis of overall survival after surgery (*n* = 50)**

*HR* hazards ratio; *CI* confidence interval; *AJCC* American Joint Committee on Cancer

* *P* < 0.05

**Supplementary Table 3 The interacting miRNAs of circHECTD1 predicted by CircInteractome.**

| **CircRNA (top) - miRNA (bottom)** | **CircRNA (top) - miRNA (bottom) pairing** |
| --- | --- |
| hsa_circ_0031485 (5' ... 3')  [hsa-miR-1256](http://www.mirbase.org/cgi-bin/mirna_entry.pl?acc=hsa-miR-1256) (3' ... 5') | AAACAGAUGGGGAAAAAUGCCAG                 \|\|\|\|\|\|    UCGAUCACUCUUCAGUUACGGA |
| [hsa_circ_0031485](http://www.circbase.org/cgi-bin/singlerecord.cgi?id=hsa_circ_0031485) (5' ... 3')  [hsa-miR-1229](http://www.mirbase.org/cgi-bin/mirna_entry.pl?acc=hsa-miR-1229) (3' ... 5') | CAAGGGUGGAGACAUGUGAGAAU        \|\|\|\|         \|\|\|\|\|\|   GACACCCUCCCGUCAC----CACUCUC |
| [hsa_circ_0031485](http://www.circbase.org/cgi-bin/singlerecord.cgi?id=hsa_circ_0031485) (5' ... 3')  [hsa-miR-1292](http://www.mirbase.org/cgi-bin/mirna_entry.pl?acc=hsa-miR-1292) (3' ... 5') | CAGAGACGUCUAGUACGUUCCCA                    \|\|\|\|\|\|\|    GUCGCAGACGGCCUUGGGCAAGGGU |
| [hsa_circ_0031485](http://www.circbase.org/cgi-bin/singlerecord.cgi?id=hsa_circ_0031485) (5' ... 3')  [hsa-miR-136](http://www.mirbase.org/cgi-bin/mirna_entry.pl?acc=hsa-miR-136) (3' ... 5') | CUUUAUGGCACUGUAAAUGGAGU                \|\|\|\|\|\|\|  AGGUAGUAGUUUUGUUUACCUCA |
| [hsa_circ_0031485](http://www.circbase.org/cgi-bin/singlerecord.cgi?id=hsa_circ_0031485) (5' ... 3')  [hsa-miR-142-3p](http://www.mirbase.org/cgi-bin/mirna_entry.pl?acc=hsa-miR-142-3p) (3' ... 5') | UGCCAGUGGACAAACACACUACC                   \|\|\|\|\|\|\|     AGGUAUUUCAUCCUUUGUGAUGU |
| [hsa_circ_0031485](http://www.circbase.org/cgi-bin/singlerecord.cgi?id=hsa_circ_0031485) (5' ... 3')  [hsa-miR-142-5p](http://www.mirbase.org/cgi-bin/mirna_entry.pl?acc=hsa-miR-142-5p) (3' ... 5') | AUUAUCUGGAUUCGAACUUUAUG                \|\|\|\|\|\|\|    UCAUCACGAAAGAUGAAAUAC |
| [hsa_circ_0031485](http://www.circbase.org/cgi-bin/singlerecord.cgi?id=hsa_circ_0031485) (5' ... 3')  [hsa-miR-335](http://www.mirbase.org/cgi-bin/mirna_entry.pl?acc=hsa-miR-335) (3' ... 5') | CAACUGCAACUUGGCCUCUUGAU                 \|\|\|\|\|\|   UGUAAAAAGCAAUAACGAGAACU |
| [hsa_circ_0031485](http://www.circbase.org/cgi-bin/singlerecord.cgi?id=hsa_circ_0031485) (5' ... 3')  [hsa-miR-515-3p](http://www.mirbase.org/cgi-bin/mirna_entry.pl?acc=hsa-miR-515-3p) (3' ... 5') | CAGCCCACAGGGAGAAGGCACUG                  \|\|\|\|\|\|\|     UUGCGAGGUUUUCUUCCGUGAG |
| [hsa_circ_0031485](http://www.circbase.org/cgi-bin/singlerecord.cgi?id=hsa_circ_0031485) (5' ... 3')  [hsa-miR-519e](http://www.mirbase.org/cgi-bin/mirna_entry.pl?acc=hsa-miR-519e) (3' ... 5') | CAGCCCACAGGGAGAAGGCACUG                  \|\|\|\|\|\|\|     UUGUGAGAUUUUCCUCCGUGAA |
| [hsa_circ_0031485](http://www.circbase.org/cgi-bin/singlerecord.cgi?id=hsa_circ_0031485) (5' ... 3')  [hsa-miR-561](http://www.mirbase.org/cgi-bin/mirna_entry.pl?acc=hsa-miR-561) (3' ... 5') | CAUUAUCUGGAUUCG-AACUUUAU         \|\|\|\|    \|\|\|\|\|\|    UGAAGUUCCUAGAAUUUGAAAC |
| [hsa_circ_0031485](http://www.circbase.org/cgi-bin/singlerecord.cgi?id=hsa_circ_0031485) (5' ... 3')  [hsa-miR-593](http://www.mirbase.org/cgi-bin/mirna_entry.pl?acc=hsa-miR-593) (3' ... 5') | GCUAAUCUUAGACGGCAGAGACG                   \|\|\|\|\|\|\|         UCUUUGGGGUCGUCUCUGU |
| [hsa_circ_0031485](http://www.circbase.org/cgi-bin/singlerecord.cgi?id=hsa_circ_0031485) (5' ... 3')  [hsa-miR-648](http://www.mirbase.org/cgi-bin/mirna_entry.pl?acc=hsa-miR-648) (3' ... 5') | AUGCCAGUGGACAAACACACUAC    \|\|\|\|\|\|      \|\|\|\|\|\|     UGGUCACGGGAC-GUGUGAA |
